# Immunocompetent and Immunodeficient Mouse Models for Enterovirus 71 Pathogenesis and Therapy

**DOI:** 10.3390/v10120674

**Published:** 2018-11-28

**Authors:** Chiaho Shih, Chun-Che Liao, Ya-Shu Chang, Szu-Yao Wu, Chih-Shin Chang, An-Ting Liou

**Affiliations:** 1 Institute of Biomedical Sciences, Academia Sinica, Taipei 11529, Taiwan; jfliao@ibms.sinica.edu.tw (C.-C.L.); daph7516@gmail.com (Y.-S.C.); guguma126@yahoo.com.tw (S.-Y.W.); sony721108@hotmail.com (C.-S.C.); sh3333@msn.com (A.-T.L.); 2 Institute of Microbiology and Immunology, National Yang-Ming University, Taipei 11221, Taiwan

**Keywords:** Enterovirus 71, EV71, EV-A71, animal models, pathogenesis, therapy

## Abstract

Enterovirus 71 (EV71) is a global health threat. Children infected with EV71 could develop hand-foot-and-mouth disease (HFMD), encephalitis, paralysis, pulmonary edema, and death. At present, no effective treatment for EV71 is available. We reviewed here various mouse models for EV71 pathogenesis and therapy. Earlier studies relied on the use of mouse-adapted EV71 strains. To avoid artificial mutations arising de novo during the serial passages, recent studies used EV71 clinical isolates without adaptation. Several human receptors for EV71 were shown to facilitate viral entry in cell culture. However, in vivo infection with human SCARB2 receptor transgenic mice appeared to be more limited to certain strains and genotypes of EV71. Efficacy of oral infection in these transgenic models is extremely low. Intriguingly, despite the lack of human receptors, immunodeficient neonatal mouse models can still be infected with EV71 clinical isolates via oral or intraperitoneal routes. Crossbreeding between SCARB2 transgenic and stat1 knockout mice generated a more sensitive and user-friendly hybrid mouse model. Infected hybrid mice developed a higher incidence and earlier onset of CNS disease and death. Different pathogenesis profiles were observed in models deficient in various arms of innate or humoral immunity. These models are being actively used for antiviral research.

## 1. Introduction

**Figure 1:**
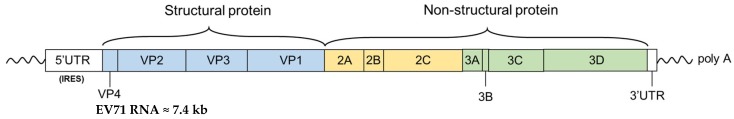
The genomic organization of EV71. The plus-strand RNA of EV71 genome encodes a single polyprotein precursor. Upon proteolytic processing by virus-encoded proteases 2A and 3C, this polyprotein precursor converts into several smaller mature products, including 4 structural proteins (VP1-VP4 capsid protein) and 7 non-structural proteins (2A, 2B, 2C, 3A, 3B, 3C and 3D). 3B encodes a VPg protein, which is covalently bound to the 5′ end of the RNA genome. 3D encodes RNA-dependent RNA polymerase. IRES: internal ribosome entry site. This cartoon illustration is based on gt B5 clinical isolates in Taiwan [4,5]. However, it is representative of the general genomic structure of all genotypes isolated elsewhere.

Enterovirus 71 (EV71 or EV-A71) was first isolated from the stool of encephalitis patients in California in 1969 [1]. It is a positive-strand RNA virus, belonging to the *Picornaviridae* family [2,3]. The genomic structure of EV71 is as outlined in Figure 1. Similar to poliovirus and other plus-strand RNA viruses, it contains an IRES element, which drives the translation of a polyprotein from a 7.4 kb genomic mRNA template. Upon protease cleavage, this polyprotein precursor can then convert into various smaller-size structural and non-structural proteins, leading to capsid assembly. As the focus in this review is about pathogenesis and animal models, readers interested in the EV71 replication cycle are advised to consult other reviews in literature.

Adults infected with EV71 could develop subclinical or cold-like syndrome. However, children younger than five years of age could develop hand-foot-and-mouth disease (HFMD) and herpangina. In severe cases, EV71-infected children could develop aseptic meningitis, encephalitis or myocarditis [6]. In a long-term follow-up study, EV71-infected patients with CNS involvement and/or cardiopulmonary failure was associated with delayed neurodevelopment and reduced cognitive functioning [7]. Unfortunately, some patients were still not saved due to pulmonary edema and/or heart failure [8].

Although EV71 was first discovered in the US decades ago, recent major outbreaks occurred frequently in the Asia-Pacific region, including Taiwan, Singapore, China, Malaysia, and Japan [6,9,10,11,12]. China alone reported 1894 deaths between 2008 and 2011 [12]. Sporadic cases were also reported from Europe [13,14], Russia [15] and Australia [16], indicating that EV71 is a global health threat. Like poliovirus in the last century, EV71 is emerging rapidly as another pathogenic picornavirus for children in this century.

Intravenous immunoglobulin (IVIG) is a therapeutic strategy for EV71-associcated brainstem encephalitis [17]. However, an antibody-dependent enhancement (ADE) phenomenon was observed in EV71-infected patients [18,19,20,21], suggesting that IVIG may not be the most adequate treatment in clinical practice. Milrinone, a phosphodiesterase inhibitor, was used to treat patients with EV-71 induced pulmonary edema (PE). While Milrinone is not an antiviral per se, mortality was significantly decreased in the Milrinone-treated group, suggesting that it could be a useful therapeutic drug for EV71-associated PE [22].

To investigate the pathogenesis and therapy for EV71 infection, we will need both in vitro and in vivo models of EV71 infection. In this review, we cataloged here from the literature a total of 16 cell lines (Table 1) and 22 animal models (Table 2), which had been tested for EV71 infection and pathogenesis. We compare below their respective advantages, limitations, and caveats in using these models.

## 2. In Vitro Propagation Systems

The most common cell line for EV71 infection in culture is the human RD cell line of rhabdomyosarcoma origin [23]. This RD cell line can produce a high yield of virus particles and thus is often used for amplification of EV71 in animal experiments [4] and studies on viral structure [56]. In addition to RD cells, various cell lines used in poliovirus infection, such as HeLa and HEK293, are also susceptible to EV71 infection and transfection [36,37]. These cell lines were used as a platform to investigate the function of viral protein. From clinical samples, EV71 protein can be identified in brainstem [6,57], and thus EV71 is considered as a neurotropic virus [1]. Several neuronal cell lines, including SK-N-SH, NSC-34 and SF268, had been used in a number of studies, such as dissecting the viral life cycle, host response to EV71 [33], phylogenetic analysis [24], and anti-viral drug testing [34]. In natural infection, EV71 is transmitted through an oral-fecal route [58]. Some previous studies therefore focused on the GI tract immune responses to viral infection and replication kinetics in the intestinal epithelial cells [25,26].

Using the Jurkat cell line and an expression cloning strategy by panning, Nishimura et al. identified human P-selectin glycoprotein ligand-1 (PSGL-1; CD162) as a functional receptor on leukocytes for EV71 viral entry [59]. However, PSGL-1 cannot be the only receptor for EV71, since PSGL-1-independent infection in non-leukocytes was also observed. Yamayoshi et al. reported that human scavenger receptor class B, member 2 (SCARB2, also known as lysosomal integral membrane protein II or CD36b like-2) is another receptor for EV71 [60]. The exogenous expression of human SCARB2 converted non-permissive cell lines to instead support EV71 propagation and develop cytopathic effects. EV71 infection can be interfered by the antibody to SCARB2 or by soluble SCARB2. Several alternative receptors for EV71 have also been reported [61,62,63,64].

In addition to Jurkat cells, THP-1 and human PBMC had been used to study the innate immunity in EV71 infection [37]. Many other cell lines contributed to the studies on EV71 molecular virology, virus-host interactions, or drug screening (Table 1). So far, the human rhabdomyosarcoma cell line RD appeared to be the most commonly used gold standard in literature, in part due to its high yield production of different EV71 genotypes [65]. As more and more laboratories are using the same RD cell line (e.g., from ATCC), it would be easier to cross-compare results from different laboratories. Since these cell line models are limited one way or the other in their resemblance to human natural infection, an alternative approach is needed to validate the findings from the cell culture system. In this regard, animal modeling can provide more complete and convincing evidence for our understanding on EV71 in vivo infection and pathogenesis.

## 3. In Vivo Animal Models

Experimental results learned from the cell culture system need to be validated in animal models and human clinical trials. Table 2 summarizes different EV71 mouse models reported in literature so far. Overall, one thing in common in these different models is their frequent use of limb paralysis and mortality for scoring disease severity. This is in part due to the fact that paralysis and death are more apparent, objective, and convenient phenotypes than other parameters, such as ataxia, or pulmonary edema. Another common feature is the requirement of a neonatal host age for productive in vivo infection, in particular with clinical isolates. Although the exact mechanism for this young age requirement remains unclear, it is technically demanding and not user-friendly, when the newborns are too small and fragile to handle experimentally. When tissue cytokine/chemokine profiles were examined in these different EV71 mouse models, they were often found to share some inflammatory cytokines in common, such as IL1β, IL6, IL10, IP10, MCP-1, IFN-α, and IFN-γ, etc. [4,52,53]. For neuropathogenesis models, the distribution of viral antigens is also quite similar among different models, which mimics human cases with fatal encephalomyelitis. For example, inflammation and viral antigens in human cases were observed mainly in the spinal cord, brainstem, hypothalamus, cerebellar dentate nucleus, and cerebrum [66]. In the *stat-1* KO model, VP1 was strongly expressed throughout the entire spinal cord, including the anterior horn and the gray matter [4]. Furthermore, VP1 in the pons of the brainstem, but not in the cerebral cortex, supports the concept of retrograde axonal transport from the peripheral nerves to the spinal cord ascending to the CNS. This result is supported from the mouse models infected with mouse-adapted EV71 strains [67,68] and poliovirus [69].

As summarized above, there is a high degree of similarity among EV71-infected human patients and mouse models. However, there are also important differences among these mouse models, which will need to be discussed individually.

### 3.1. Mouse Models Dependent on Mouse-Adapted EV71 Strains

In general, adult humans are resistant to EV71 infection and pathogenesis. Similarly, adult mice cannot be easily infected with EV71 clinical isolates. To solve this problem, researchers developed mouse-adapted strains of EV71, such as MP4, in an ICR mouse model [21,27,44,45,46]. MP4 was generated through i.p. injection of the parental EV71 TW/4643/98 into one-day-old ICR mice. Adapted EV71 recovered from the brain tissue was re-used to repeat the serial injection-adaptation cycles. EV71 recovered from the fourth passage was designated as MP4 [27]. Comparing to its parental EV71 isolates, MP4 is more cytotoxic with a larger plaque size. Because EV71 is a RNA virus with a quasi-species nature, mouse-adapted strains tend to accumulate many artificial mutations from the serial passages. One legitimate concern is that these adaptive mutations cannot really be found in human natural infection. In addition to MP4, mouse adapted strains were also generated in the immunodeficient NOD/SCID mouse model [55]. Comparison of the amino acid sequences between clinical isolates and mouse adapted strains revealed major differences in structural proteins, such as VP1, VP3, and protease 2A [55]. Therefore, it always remains uncertain whether results obtained from using the adapted strain of EV71 can be automatically extended to human natural infection with the clinical isolates.

### 3.2. Immunodeficient Mouse Models

Host immune system plays a critical role against viral infection [70]. Immunodeficient mice, even without bearing any human receptors for viral entry, can support infection with EV71 clinical isolates, instead of relying on mouse-adapted strains. Innate immunity, such as IFNs, is required for protection from EV71 infection and pathogenesis [71,72]. AG129 mice, which is deficient in both α/β and γ interferon receptors, developed limb paralysis and death, when infected with a non-mouse-adapted EV71 via i.p. or oral routes. Viral protein was detected in CNS in both i.p. and oral infection [53]. Like mouse AG129, mouse A129 (deficient in α/β interferon receptor) can also be infected with mouse-adapted strain of EV71 [54]. Moreover, mouse G129 deficient in γ interferon receptor developed limb paralysis via an i.p. route with clinical isolates of EV71 [4] (Table 2). Another host factor *stat-1* is a key transcription factor in IFN signaling. The *stat-1* knockout (KO) mice can be successfully infected with both genotype B and C clinical isolates of EV71 [4,5]. Infected *stat-1* KO mice developed hindlimb paralysis with abundant viral protein in the CNS system, supporting an important role of IFN signaling in protecting CNS from EV71 infection and pathogenesis.

In addition to innate immunity, humoral immunity also plays an important role in EV71 infection and pathogenesis. For example, injection with an EV71-specific neutralizing antibody before or after infection, can strongly reduce lethality and tissue viral loads of a mouse-adapted strain M2 in a B cell-deficient C57BL/6-derived mouse model [44]. In another example, NOD/SCID mice deficient in T and B lymphocytes can be infected with a mouse-adapted strain of EV71 [55]. However, since mouse-adapted EV71 can infect even immunocompetent mice [27], it was unclear if the missing T and B lymphocytes in the NOD/SCID mouse model were really important for protection from mouse-adapted EV71 [55]. This issue was addressed in one study when NOD/SCID mice were inoculated with EV71 clinical isolates and developed limb paralysis via either an i.p. or oral route. Although limb paralysis has been used as a common marker for scoring EV71 pathogenesis in mouse modeling, acute flaccid paralysis does not occur as frequently as hand-foot-and-mouth disease (HFMD) in human natural infection. Interestingly, in the NOD/SCID model infected with clinical isolates, an HFMD-like phenotype with skin rash was observed, for the first time in mouse models [4]. Spleen atrophy was found in some EV-71 infected children [57,73], and was also noted in this NOD/SCID model infected with clinical isolates [4]. Moreover, strong VP1 staining and severe inflammation were apparent in the paralyzed muscle. On the other hand, no viral protein and pathology was ever detected in the CNS in EV71-infected NOD/SCID mice. It is worth mentioning here that in this NOD/SCID model, kinetic analysis in a time course experiment revealed more delayed clearance of non-infectious viral RNA relative to the clearance of infectious viral particles [4]. Furthermore, the inflammatory IL-23/IL-17 axis appeared to be activated in the infected NOD/SCID model. It remains to be seen whether these intriguing phenomena are idiosyncratic to the NOD/SCID model, or they can be generalized to other mouse models or human patients.

Interferon-gamma-inducible protein-10 (IP-10) is a highly expressed chemokine in EV71-patients [74]. IP-10 knock-out mice infected with mouse-adapted EV71 exhibited a higher mortality rate than the wild type control mice, suggesting a protective role of IP-10 in EV71 infection and pathogenesis [49].

While the immunodeficient models are very useful for studying pathogenesis and evaluating efficacies of drug and vaccine candidates, it is not always a good system for testing immune modulators in therapeutic research. In addition, it is not as direct a platform for vaccine research either, since it can only be used for comparing potencies of anti-EV71 neutralizing antibodies raised against different vaccine candidates.

### 3.3. Transgenic Mouse Models

Recently, many cellular receptors have been proposed for EV71 viral entry in vitro, including hSCARB2 (human scavenger receptor class B) [60], PSGL1 (P-selectin glycoprotein ligand 1 [59], annexin II [61], and nucleolin [62]. These receptors facilitated EV71 infection in non-susceptible cell lines, such as mouse fibroblast L929. However, transgenic (Tg) mice expressing human PSGL-1 failed to support infection with EV71 clinical isolates [50]. Among these reported cellular receptors, only human SCARB2 (hSCARB2) has so far been shown to support EV71 infection in vivo.

There are two different hSCARB2 Tg mouse models in literature (Table 3) [51,52]. In the EF-1a-hSCARB2 model, EF-1a promoter is used to drive hSCARB2 expression in the C57B/6 mouse background [52]. In the SC2-hSCARB2 model, the native hSCARB2 promoter in a human SCARB2 BAC clone drives the transgene expression in the C57B/6 mouse background [51]. Both models can be infected with EV71, develop limb paralysis, and detect viral protein in multiple tissues, including muscle (muscle-tropism) and the CNS system (neurotropism). On the other hand, these two models cannot be easily compared with each other due to a number of differences (Table 3): (1) virus strains and genotypes (B4, B5, C2, C4 vs. Isehara); (2) host age at virus inoculation (1- or 7-day-old vs. 3-wk-old); (3) virus dose (PFU vs. TCID_50_); (4) inoculation route (s.c. vs. i.c., i.v., i.p.); (5) assays for readout (individual score of paralysis & death vs. combined score of ataxia, paralysis, and death). In addition to the Isehara strain (gt C2), other genotypes of EV71 were tested in the SC2-hSCARB2 model [51]. However, since the non-Tg wild type mouse control was included only in the study using the Isehara strain, we listed here in Table 3 only the results based on the Isehara strain.

In the pEF-1a-hSCARB2 transgenic mouse model, limb paralysis rate cannot be differentiated between Tg and non-Tg control mice, when EV71 gt B4 or gt C4 are used (Table 3) [52]. However, mortality rates between Tg and non-Tg are different for gt C4; and the incidences of earlier symptoms are different between Tg and non-Tg, when gt B4 was used. Despite the limitation in host age and susceptibility to certain EV71 genotypes in this pEF-1a-hSCARB2 transgenic model, it had been used in evaluating the efficacy of EV71 vaccine or adjuvant [75]. The Isehara strain behaved like a strain with a high degree of virulence. For other viral isolates, such as BrCr, SK-EV006, and C7, much higher viral doses were required for developing neurological symptoms in the hSCARB2-Tg10 mouse model. In fact, even subcutaneous infection of the neonatal non-Tg control mice with the Isehara strain caused paralysis [51]. In addition to the CNS, viral antigens were detected in skeletal muscle of both non-Tg and Tg mice. In this regard, the hSCARB2 Tg mice are similar to the NOD/SCID mice in supporting the replication of muscle-tropic EV71.

Neither model of these hSCARB2 Tg mice has so far been shown to support efficient oral (intragastric) infection, which is a major route for transmission in children [51,52]. Before these models are shown to be susceptible to a broader source of different clinical isolates and genotypes, it is a caveat to carefully choose the best EV71 strains in the experimental design based on the hSCARB2 transgenic model.

### 3.4. A Hybrid Mouse Model

As discussed above, both hSCARB2 receptor and immune deficiency can contribute to efficient EV71 infection and pathogenesis. Therefore, it is possible that a combination of a transgenic hSCARB2 and host immune deficiency might further enhance infection efficiency. Indeed, a new hybrid mouse model was generated by cross-breeding hSCARB2 Tg and *stat-1* KO mice. Comparing to its parental hSCARB2 Tg or *stat-1* KO mice, this hybrid mouse model is more user-friendly. For example, at 2-week-old age, it can still be i.p. infected with different genotypes of EV71 at 1000-fold lower titer (pfu) than the parental mice [5]. EV71-infected hybrid mice exhibited high density of viral protein in the CNS, such as mid-brain and spinal cord. Like the *stat-1* KO model, although EV71-infected hybrid mice developed limb paralysis, neither viral RNA nor protein was ever detected in the muscle. It suggests that paralysis originated solely from the CNS injury, rather than myositis and muscle destruction. This new hybrid (hSCARB2 Tg/*stat-1* KO) model could serve as a user-friendly platform for evaluating drug or vaccine efficacy [5].

Although this hybrid mouse model is a far more sensitive system for studying EV71-associated neuropathogenesis and antiviral therapy, it is deficient in interferon signaling. Therefore, it is not a good model for studying the therapeutic potential of immune modulators.

## 4. Pathogenesis Mechanism

Disease manifestations in EV-71 infected children are very diverse, ranging from subclinical infection, common cold-like syndromes, hand-foot-and-mouth disease, to uncomplicated brainstem encephalitis, severe dysregulated autonomic nerve system, fatal pulmonary edema, and cardiopulmonary collapse. To date, while neurological disorders can be modeled in *stat-1* KO mice, hybrid mice, and hSCARB2 Tg mice, pulmonary edema and cardiopulmonary failure remain to be recapitulated in EV71 mouse models. Because viral protein has never been detected in patients’ cardiomyocytes, it is generally believed that cardiopulmonary failure is caused indirectly by the dysregulated autonomic nerve system in the infected brainstem. Depending on the combination of both viral and host factors, current EV71 animal models could develop a wide variety of pathogenesis similar to human infection. The viral factors include the infectious dose of EV71, genotypes, mutations, and possibly, sequence heterogeneity (quasispecies). The host factors include the innate and adaptive immunity, routes of virus inoculation, and the critical host age receiving viral infection.

### 4.1. Viral Determinants of Virulence

In the case of viral factors, both viral load and sequence variations of different EV71 isolates could significantly contribute to different degrees of virulence and clinical outcome. In the 1970s, EV71 epidemics occurred in North America and Europe (genotype A, B1, and B2) and showed a higher percentage of paralysis than the dermatological symptoms [76,77]. In contrast, clinical reports in Asia-Pacific after the 1980s (other than genotype A, B1, and B2) observed both dermatological and neurological symptoms [3,21,45]. Comparison of these results suggested that differences in ethnic groups or/and genotypes of EV71 may have different sequelae. Sequence analysis identified some nucleotide or amino acid differences between fatal and non-fatal cases [78,79,80]. For example, it has been shown that point mutations at amino acid 145 of VP1 or amino acid 149 of VP2 could determine whether neonatal mice can or cannot be successfully infected with a mouse-adapted EV71 strain [55,81]. In addition to the coding sequences, adaptive mutations at the IRES region also affected the virulence in mice [82]. Finally, tissue tropism could also be associated with viral sequence variations. Sequence comparisons of different EV71 isolated from respiratory, gastrointestinal, CNS, and blood specimens, revealed nucleotide differences at three different positions. Further study showed that amino acid 97 of VP1 was critical for neurotropism [83]. Both amino acid 97 and amino acid 145 of the capsid protein VP1 were further investigated by two different research groups for their contribution to pathogenesis in hSCARB2 Tg and non-Tg mouse models, respectively [84,85]. VP1 mutant L97R was thought to be more virulent, because it acquired better binding to heparan sulfate on the target cells [85]. Heparan sulfate is a common attachment receptor for many viruses, including EV71 [63]. In contrast, VP1 mutant 145G was thought to be less neurovirulent than VP1 mutant 145E, due to its better binding to heparan sulfate than mutant 145E [84]. It was proposed that VP1-145G virus is preferentially adsorbed by heparan sulfate attachment receptors on an excessive number of non-target cells during circulation in vivo, leading to trapping and attenuation of mutant VP1-145G. In summary, it would have to be resolved in the future, whether heparan sulfate binding property of EV71 capsid protein VP1 and in vivo viral virulence, are positively or negatively correlated.

### 4.2. Host Factors

In this section, we will focus our scope on only those host factors which were investigated in animal models. In one previous study [4], mouse models with different immunogenetic backgrounds were i.p. infected with the same clinical isolates of EV71, and different disease outcomes and viral tropisms were observed. For example, in the NOD/SCID mouse model infected with an EV71 clinical isolate (gt B5), EV71 was predominantly muscle tropic, with severe atrophy and inflammation in the paralyzed hindlimb muscle. In contrast, in the *stat-1* KO and the hybrid mouse models, EV71 behaved neurotropic with VP1 signals in both brain and spinal cord [4,5]. In the *stat-1* KO model, VP1 protein was often enriched in the Purkinje layer of cerebellar cortex, pons, brain stem, and spinal cord. IBA-1+ microglia cells were found to be amplified, indicating a high degree of neuronal injury. In the hybrid mouse model, Nissl staining of spinal cord sections revealed abnormal neurons with extensive vaccuoles. Unlike the NOD/SCID model, neither VP1 signal nor inflammation was ever detected in the paralyzed muscle in *stat-1* KO or hybrid mouse models. Since *stat-1* is an upstream transcription factor important for interferon signaling, *stat-1* or interferon deficient CNS appeared to favor neurotropic EV71. By contrast, limb muscles deficient in T and B cells appeared to favor muscle-tropic EV71.

In human clinical cases, since EV71 infection in Europe and Asia-Pacific had different clinical features, the difference in their respective ethnic (and immunogenetic) backgrounds (Caucasian vs. Asian) could be responsible for their different disease manifestations [6,76,86]. Cytotoxic T-lymphocyte antigen-4 (CTLA-4) is an important regulator of T-cell cytotoxicity and tolerance. The polymorphism of CTLA-4 was found to be associated with encephalitis in EV71-infected children [87]. Other genetic polymorphisms, such as human leukocyte antigen-A33 (HLA-A33) [88], tumor necrosis factor-α (TNF-α), and interferon-α receptor 1 (IFNAR1) [89,90], were also found to be associated with disease severity in EV71 patients. In summary, both viral and host factors are important for EV71 immunopathogenesis.

## 5. Therapy

EV71 vaccination should be an effective way to protect children from infection. While inactivated whole virus of EV71 appears to be a good vaccine candidate [91,92,93,94], it is unclear if it can be used for all age groups of children. Therapeutics against EV71 remains an urgent need. We summarized here in Table 4 a list of candidate therapeutics, which had been tested in patients or animal models. We categorized these candidate compounds into four different groups according to their approaches.

Group 1 relied on passive immunization with anti-EV71 neutralizing sera raised against recombinant VP1 protein, virus-like particles (VLP), formaldehyde-inactivated whole virus, and IVIG from human source [ref cited in Table 4]. Although IVIG were used in clinical practice [17], the possibility of antibody-dependent enhancement of EV71 must be seriously considered [19,20,21]. Another important issue is whether these immune sera or antibodies can efficiently and broadly cross protect against EV71 of different genotypes. Mouse monoclonal antibodies (mAb) need to be humanized and re-tested in animal models in the future. Immune escape variants could be selected during mAb treatment.

Group 2 includes immune modulators, such as interferon (IFN-α) or TLR agonists. Pre-treatment with Poly (I:C) or co-treatment with mouse IFN and EV71, can reduce the death rate in EV71-infected mice [45]. However, some of these studies in Group 2 are prophylactic rather than therapeutic. Oral intake of GS-9620 (a TLR7 agonist) inhibited viral replication and increased the survival rate of ICR mice infected with a mouse-adapted strain MP10 [95]. In clinical trials, IFN-α can decrease the fever and viral load in mild HFMD patients [72,96]. Altogether, activation of IFN signaling could be a potential therapy for EV71 infection.

Group 3 includes anti-inflammation treatments, such as anti-IL6 Ab, and complement inhibitors [38,97]. Although compounds in Group 3 may not have any direct antiviral effect, they can help reduce inflammatory injury by excessive cytokines.

Group 4 includes various compounds, whose mechanisms either remain unclear at present, or do not belong to the above Group 1–3 [22,98,99,100,101]. For example, Milrinone is supposed to help maintain the level of catecholamine [22]. As a side note, it is worth mentioning that disinfectants against EV71 have also been investigated. While disinfectants have no therapeutic value, solutions of chlorine dioxide (ClO_2_) [102] and methylene blue (photosensitizer) [103] can inactivate EV71.

## 6. Conclusions

In 2018, there are many issues that need to be solved in the modeling of EV71 infection and pathogenesis. First, while the immune deficient NOD/SCID mice offer an efficient model for oral infection with EV71 clinical isolates, one remaining challenge is to establish an oral infection system in an immune-competent mouse model. Second, another mystery is the requirement of a neonatal age for productive EV71 infection and pathogenesis. It is widely speculated that some kind of age-related immune maturation could confer the resistance to pediatric infections, such as EV71. However, if so, the exact nature of such an age-dependent arm of the immune system remains elusive. It cannot be excluded that a putative EV71 receptor could be expressed in muscle or nerve only transiently at the neonatal age, but rapidly turned off at an older age during mouse development. Third, there is an urgent need for FDA-approved antivirals against the frequent EV71 epidemics in Asia and worldwide. Lessons that we learned from EV71 should be useful for other picornaviruses closely related to EV71, such as the most recent D68 virus that emerged in North America a few years ago.

## Figures and Tables

**Table 1:** Summary of 16 cell lines used for EV71 in vitro infection.

Cell Line	Species	Tissue Origin	EV71 Genotype	CPE ^#^	References
RD	Human	Rhabdomyosarcoma	A, B, C, D	**+**	[23,24,25,26,27,28,29,30]
SK-N-SH		Neuroblastoma	C2, D	**+**	[24,27]
SH-SY5Y		Neuroblastoma	C4	**+**	[31,32]
NSC-34		motor neuron cell line	B2, B4 & C2	**−**	[33]
SF268		glioblastoma	*	**+**	[34]
Neuro-2A	Mouse	Neuroblastoma	D	**+**	[24,35]
HT29	Human	intestinal epithelial cell	B4, C	**+**	[25,26,28]
Caco-2		colorectal carcinoma	B, C2	**+**	[27,29]
C2BBel		mature intestinal epithelial cell (derived from Caco-2)	C	**+**	[28]
HeLa	Human	cervical carcinoma	EV71:BS (Accession NO KF514878), C4	**+**	[35,36]
HEK293T	Human	embryonic kidney	EV71:BS (Accession NO KF514878), C4	**+**	[35,37]
THP-1	Human	monocytic cell line	C2, C4		[19,37]
human PBMC			C2		[19]
Vero	Monkey	African green monkey kidney	A, EV71:BS (Accession NO KF514878)	**+**	[30,35]
COS-7		African green monkey kidney (SV40 transformed)	EV71:BS (Accession NO KF514878)	**+**	[35]
NIH/3T3	Mouse	mouse embryonic fibroblast	mouse-adapted strain (EV:TLLm)	**+**	[35]

* information not available. ^#^ CPE, cytopathic effect. Genotype D is a C4 subgenotype.

**Table 2:** Summary of 22 animal models used for EV71 in vivo infection.

Mouse Strain	Host Age ^@^	Route	Genotype	Titer/Mouse	Phenotype	Virus-Replicating Tissue	Reference
Balb/C	1-day-old	i.p.	B4	1 × 10^4^ PFU	limb paralysis & death	intestine	[38]
	2-day-old	i.p./i.c.	C4	10^3^ TCID50	*	brain, liver and intestine	[39]
	5-day-old	i.p.	C4 (mouse adapted strain)	10^4^ TCID50	limb paralysis & death	spleen, heart, liver, muscle and brain	[40]
	7-day-old	i.m. i.p. i.c.	B3 (CHO cell adapted strain; CHO-26M) (mouse adapted strain; MP-26M)	10^3^ TCID50	death	muscle (CHO-26M) blood, spleen, heart, liver, muscle and brain (MP-26M)	[41]
	9-day-old	i.p.	C4	10^4.5^ TCID50	limb paralysis	skeletal muscle, spinal cord, brainstem, lung, and jejunum	[24]
ICR	1-day-old	oral	C (mouse adapted strain)	10^6^–10^7^ PFU	paralysis & skin lesion	skin, intestine and spinal cord	[42]
	1-day-old	i.p.	C4 (Fuyang-0805a)	2 × 10^5^ TCID50	none	muscles, intestines, lungs, and brain	[43]
	7-day-old	oral	C (mouse adapted strain M2)	5 × 10^6^ PFU	death	*	[44]
	7-day-old	i.p.	C2 (mouse adapted strain MP4)	2.5 × 10^6^ PFU	death	brain, spinal cord, lung and muscle	[21]
	3-day-old	i.p.	C2 (clinical isolate)	4 × 10^6^ PFU	death	*	[45]
	7-day-old	oral	C2 (mouse adapted strain MP4)	5 × 10^5^ PFU	death	*	[45]
	12–14-day-old	i.p.	C (mouse adapted strain M2)	5 × 10^4^–10^5^ PFU	paralysis & death	*	[46]
	14-day-old	i.p.	B3 (mouse adapted strain)	100 CCID50	paralysis	brain and muscle	[47]
	14-day- & 28-day-old	i.p. i.m. oral s.c.	B3 (mouse adapted strain)	10^5^ CCID50	paralysis	mainly in CNS	[48]
C57BL/6	9-day-old	oral	C (mouse adapted strain M2)	3 × 10^7^ PFU	*	*	[44]
C57BL/6J	14-day-old	i.p.	C (mouse adapted strain M2)	3 × 10^5^ PFU	hunched posture, paralysis & death	brain	[49]
PSGL-1 Tg	10-day-old	i.p.	C4 (clinical isolate)	10^8^ TCID50	no disease onset	brain, spinal cord and skeletal muscle	[50]
			C4 (mouse adapted strain MP10)	5 × 10^6^ TCID50	hindlimb paralysis	*	
hSCARB2 Tg	3-week-old	i.v.	C (Isehara strain)	10^2–6^ TCID50	ataxia, paralysis & death	CNS	[51]
		i.c.	A, B and C	10^2–6^ TCID50			
		i.p.	C (Isehara strain)	10^3–6^ TCID50			
		oral	C (Isehara strain)	10^6–7^ TCID50			
pEF-1α-hSCARB2	1-day-old	s.c.	B	1 × 10^7^ PFU	HFMD-like syndrome & paralysis	CNS, intestine, muscle and skin	[52]
	7-day-old	s.c.	C	3 × 10^4^ PFU			
AG129	younger than 2-week-old	i.p.	B4	5 × 10^5^–10^7^ PFU	limb paralysis & death	CNS	[53]
		oral		10^6^–10^7^ PFU			
AG129	10-week-old	i.p.	B2 (mouse adapted strain)	1.3 × 10^5^ TCID50/mL	limb paralysis & death	ND	[54]
A129							
G129	7-day-old	i.p.	B5 (clinical isolates)	10^8^ PFU	limb paralysis & death	ND	[4]
*stat-1* KO	7-day-old	i.p.	B5 (clinical isolates)	10^8^ PFU	limb paralysis & death	CNS	[4]
pSCARB2x *stat-1* KO	younger than 2-week-old	i.p.	C2 (clinical isolates)	10^6^–10^8^ PFU			[5]
NOD/SCID	3-day-old	oral	B5 (clinical isolates)	10^8^ PFU	hair loss, skin lesion, limb paralysis & death	muscle and spleen	[4]
	7-day-old	i.p.		10^7^–10^8^ PFU			
	3–4 week-old	i.v./i.c.	B1 (mouse adapted strain)	10^6.5^ CCID50		skeletal muscle and heart	[55]
*IP10* KO	14-day-old	i.p.	C (mouse adapted strain M2)	3 × 10^5^ PFU	The death rate was higher than C57BL/6J	ND	[49]

* information not available. ^@^ The age that mice infected with EV71.

**Table 3:** Comparisons between human SCARB2-transgenic and wild type non-transgenic mice in EV71 infection and pathogenesis.

Mouse Strains	Promoter	Virus Strain (Genotype)	Host Age	Virus Dose	Inoculation Route	% Earlier symptom ** (Tg vs. non-Tg)	% Limb Paralysis (Tg vs. non-Tg)	% Death (Tg vs. non-Tg)	References
pEF-1α- hSCARB2	ubiquitous EF-1 Promoter	E59 (B4)	1-day-old	3 × 10^4^ PFU	s.c.	100 vs. 57.1	18.8 vs. 14.2	0 vs. 0	[52]
		N-2838 (B5)				100 vs.75	44.4 vs. 12.5	0 vs. 0	
		5476 (C2)	7-day-old	10^7^ PFU	s.c.	Nd ^@^	100 vs. 57.1	100 vs. 0	
		N3340 (C4)				Nd ^@^	100 vs. 100	100 vs. 0	
pSC2- hSCARB2	native promoter	Isehara (C2)	3-wk-old	10^6^ TCID_50_	i.c.	N/A ^#^	100 vs. 0 *		[51]
					i.v.	N/A ^#^	89 vs. 0 *		
					i.p.	N/A ^#^	39 vs. 0 *		
					oral	N/A ^#^	5 vs. 0 *		

^@^: No disease detected; ^#^: No information available in the original paper; * combined score of ataxia, limb paralysis, and death; ** Earlier symptoms include hair loss and scurf.

**Table 4:** Therapeutic compounds tested in animal models and human patients.

Therapeutic Approaches	Drug Candidates	Drug Delivery Methods	Human or Animal Model	Host Age at Virus Inoculation	Virus Inoculation	EV71 Clinical Isolates * or Adapted Strains ^#^	Virus Genotype	Ref.
**GROUP 1** Passive immunization	bispecific anti-EV71 & CA16 Ab Bs(scFv)	i.p. injection at 24 h after inoculation	BALB/c	1-day-old	i.c.	EV71/pSVA-MP4 ^#^	B3	[104]
	VP1-specific mAb	i.p. injection at 3, 24, and 48 h after inoculation	SCARB2-Tg	1-day & 7-day-old	s.c.	E59 & 5746-TW98 *	B4 & C2	[105]
	human plasma or IVIG	in vitro pre-mix with EV71 at 37 °C for 1 h	ICR	2-day-old	i.p.	KM593929 *	C4	[106]
	human IVIG	3 i.p. injections at 4 h, 1- and 2-days after inoculation	Kunming mice	7-day-old	i.p.	AH08/06 *	C4	[107]
	EV71-specific Ab	i.p. injection 1 day before and 1 day after (ICR), or 2 and 4 days after inoculation (C57BL/6)	ICR & C57BL/6	7-day & 9-day-old	oral	M2 ^#^	C2	[44]
	adult immune sera	i.p. injection at 1hr before and 24 h after inoculation	young gerbils	21-day-old	i.p. or i.m.	strain 58301 *	C4	[108]
	human IVIG	i.v. infusion at a dosage of 1 g/kg/day for 2 days	human patients	<2-yr-old	natural infection	N/A	N/A	[17]
**GROUP 2** Immune modulators	Poly (I:C)	i.p. injection at 12 h before inoculation	ICR	3-day-old	i.p. or oral	MP4 ^#^	C2	[45]
	Ad-IFN-a	one intranasal shot within 12 h post-inoculation	BALB/c	6-day-old	i.p.	strain 41 *	B4	[71]
	antagomir -146a	i.p. injection before or after virus inoculation	C57BL/6	7-day-old	i.p.	mEV71 ^#^	B2	[109]
	GS-9620	oral uptake at 2, 26, 50 h post-inoculation	ICR	10-day-old	i.p.	MP10 ^#^	C4	[95]
**GROUP 3** Anti-inflammation	anti-IL6 Ab	i.p. co-injection of Ab and virus (day 0)	BALB/c	1-day-old	i.p.	strain 41 *	B4	[38]
	complement inhibitor (CR2-crry)	treatment post-inoculation	ICR	7-day-old	i.c.	BrCr *	A	[97]
**GROUP 4** Others	Retro-2^cycl^	i.p. injection following EV71 inoculation	BALB/c	<1-day-old	i.c.	KJ508817 *	C4	[98]
	Pleconaril	daily i.p. injection for 5 days	ICR	1-day-old	i.p.	BrCr *	A	[99]
	andrographolide sulfonate	i.p. injection once daily until 10 days post-inoculation	ICR	7-day-old	i.p.	BJ09/07 ^#^	C4	[100]
	adoptive transfer of macrophage	i.p. injection with adult macrophage at 1 day post-inoculation	ICR	10-day-old	i.p.	MP10 ^#^	C4	[101]
	Milrinone	i.v. injection within 2–6 h after admission at dosage 0.35–0.55 mg/kg/min for 72 h.	human patients	<2-yr-old	natural infection	N/A	N/A	[22]

i.p.: intraperitoneal; i.v.: intravenous; i.c.: intracranial; i.m.: intra-muscular; s.c.: subcutaneous; N/A: information not available in the original paper; * clinical isolates; ^#^ mouse-adapted strain.
